# Supplementary material for: Are carbon emissions trading and green financial instruments synergistic? -Comprehensive quantitative research based on content analysis
Source: PLoS One. 2024 Mar 7;19(3):e0298601. doi: 10.1371/journal.pone.0298601 (PMC10919601; doi:10.1371/journal.pone.0298601)
Supplement: S1 Data — (ZIP) [file pone.0298601.s001.zip › Data/地级市Z_F工作报告.zip!/║┌┴·╜¡/┤≤╟∞╩╨/└┤╫╘╣1⁄2╓┌║┼í╛┬φ┐╦╩2╛▌═°í┐_2014.docx]

　　各位代表：

　　现在，我代表市人民政府向大会报告工作，请予审议，并请市政协委员和列席会议的同志提出意见。

　　一、2013年工作回顾

　　过去一年，我们深入贯彻党的十八大和十八届二中、三中全会精神，按照市委八届三次全会要求和市九届人大二次会议部署，认真落实，努力攻坚，经济社会发展取得显著成效。

（一）经济持续健康发展。优存量上增量，促进结构调整和方式转变。经济规模稳步扩大。预计全市完成地区生产总值4332.7亿元、增长11.1%。工业在较大基数上稳健运行，二产实现增加值3434亿元、增长10.4%，其中地方规上工业增长20%。油田全力克服洪涝灾害影响，稳产原油4000万吨，生产天然气34.8亿立方米。120万吨乙烯、60万吨聚丙烯等项目逐步上产，华科15万吨C芳构化、三聚能源3万吨苯乙烯抽提等项目投产，佳昌80万片砷化镓抛光片、百森5万吨饮品等项目达产，沃尔沃整车如期下线，装备制造、食品加工、生物医药、新材料、新能源等重点产业分别增长20%、36%、15%、14%和16%。农业“三位一体”共进，一产实现增加值166亿元、增长8.1%。建设现代农业示范区100万亩，大灾之年粮食总产128亿斤；棚室果蔬产量35亿斤；猪牛羊等牲畜存栏410万头、家禽1980万只，“三退三进”规模养殖比重达到65%。服务业多元业态关联升级，三产实现增加值732.7亿元、增长14.4%。松雷购物广场、昌升国际商贸城等项目投入运营，各类专业批发市场发展到14个、成交额265亿元，商贸零售层次和商品集散功能同步提升；新华08（大庆）商品交易所实现交易额310亿元，电子商务交易额78亿元，新兴业态活力凸显；旅游、文化、物流、服务外包等行业均实现两位数增长，服务业呈现满足市域、辐射区域、多元融合发展的良好态势。经济质量不断提升。油与非油经济比例调整到42.8:57.2，非油经济比重提高3.2个百分点；三次产业比例调整到3.8:79.3:16.9，服务业比重提高1.7个百分点；全市财政收入1175.9亿元，其中地方财政收入261.2亿元，地方经济创造的财政收入占地方财政收入比重71%。工业经济效益综合指数突破900点、全省最高。万元地区生产总值综合能耗下降3.5%。经济发展动力日趋增强。建设投资千万元以上项目580个、其中超亿元279个，全市完成固定资产投资1581.8亿元、拉动经济增长6.5个百分点；社会消费品零售总额达到934.5亿元、增长16.4%，拉动经济增长1.4个百分点；社会物流总额增长23%，进出口总值增长20%。龙凤光明产业园、大同新河工业园、肇源大广工业园晋升为省级园区，以肇州杏山园区为核心区的大庆农业科技园区晋升为国家农业科技园区，全市招商到位资金313.5亿元，引进各类人才5016人，驻庆高校和科研院所与地方产业发展融合更加紧密，转化科技成果110项，高新技术产业产值2000亿元。大庆成为全国首批20家智慧城市试点示范市。

　　（二）民生保障进一步加强。“百项民生项目”扎实推进，去年初确定的具体民生实事全部落实。居民生活更加殷实。新就业5.57万人，1.4万人创业当上“老板”；企业退休人员养老金月人均增加223.7元，市区最低工资标准达到1160元、全省最高；城镇居民人均可支配收入28500元、农村居民人均纯收入13220元，分别增长13%和15%。社会保障使百姓更托底。“五险”净增6.24万人，城乡低保水平全省最高，救助对象扩展到3688户临近低保线的低收入家庭，为20万城乡低保和低收入群体发放御寒补贴7734万元，为2.8万户困难职工补助供热费4300万元；增加养老机构床位1080张，“医养结合”模式全省首创；新增城镇保障性住房1.2万套，依据村镇建设规划新改造农村泥草房1.1万户。公共交通更加便利。加密公交班次30%，延长营运时间1个小时，新开和调整线路16条，其中开通快速公交2条，新增和更新车辆373台，每万人拥有公交车17.2标台、居省内首位。通过公共场地施划、新区配建、老区完善等措施，新增停车泊位10万个。教育发展日趋均衡。新建改建公办幼儿园90所、中小学校202所，新增义务教育标准化学校8所、标 准化率达到69.2%，城乡结对帮扶学校47所，培训校长和教师1.3万人次，高考成绩进段人数创历史新高、囊括全省文理科状元；市特殊教育中心全面投用，交通技校异地新建，市建设中专获批国家实验实训基地，职业教育毕业生一次性就业率达到95%；7所高校教学与科研双丰收，八一农大在全国率先成立新农村发展研究院；大庆精神铁人精神教育内容系统写入教科书，以实现“五有”为标志全面纳入国民教育体系。医疗服务日趋方便优质。村卫生室实现全覆盖，为村卫生室和乡镇卫生院配套设备13200台件，在全省率先实现基层卫生机构标准化；城乡居民健康档案建档率85.1%，70%的社区卫生服务中心设立“健康小屋”提供免费体检；市人民医院、四县和大同区医院与空军总医院等国内4所名院联办开通远程会诊中心，会诊危急疑重病人332人次；全国714名签约名医来庆出诊1351人次、诊治病人33506人次；市三医院成为世界卫生组织心理危机干预中心大庆基地。文化生活越来越丰富。新建扩建社区文化活动中心29个、村文化活动室30个，覆盖率分别达到81.5%和73.3%；依托城乡近500个广场和沿湖休闲场地，30多万人参加广场舞等健身活动，让体育回归民众、带动全民健身的地企联合体育大会成功举办；“大庆之冬”艺术节项目获得国家级群星奖，话剧《大湿地》获得中国戏剧金狮奖，东北第四纪哺乳动物化石展入选全国十大精品陈列。社会环境安全和谐有序。食品、药品、生产等“八大领域”安全监管形成高压态势，抽检食品药品2100多批次、合格率95%，安全生产事故下降20.7%，八类主要刑事案件下降12.6%，群众安全感满意度达到98%以上；坚持“事要解决、案结事了”，重点信访积案化解率达到71.3%；建立网络问政平台，61个部门上线互动答政；领取居住证的外来人口可享受的基本公共服务扩展到12项，出台严重精神障碍患者服务管理办法，建立市特教中心自闭症儿童康复训练基地，“十类特殊人群”得到更好呵护。

　　（三）城镇化建设取得新进展。坚持规划引领、以人为核心，积极稳妥推进城镇化建设，城镇化质量位列全国地级城市第16位。市区重要功能设施进一步完善。实施重点工程97项，市妇女儿童医院等12个项目竣工，市图书馆等40个项目投用，大庆四中迁建等45个项目进展顺利；升级城区道路34条75.8公里，铁路东站和哈齐城际客专加快建设，客运西站普速车场开通，萨尔图机场年旅客吞吐量突破50万人次；新投用北城热源等4处、增加供热能力2240万平方米，4处热源实现应急连通，东城备用水源地完工蓄水，东城第一污水处理厂改造完成；开发阿斯兰小镇等高品质居住新区378万平方米，改造黎明小区等老居住区16个、153万平方米。小城镇和新农村建设扎实推进。近郊镇与城区融合建设，城关镇与重点乡镇协同发展。6个全省“百镇试点镇”加快建设，16个示范新村基本建成，实施乡镇学校、医院、“三供三治”等功能项目160项，新建改建农村公路94公里，108个村实现巷道亮化、绿化美化；县域生态旅游、粮食物流、食品加工、棚室果蔬等产业升级发展，杏山、新站、四合、连环湖等乡镇辐射力增强。城镇化促进农民稳定就业1.8万人。城乡一体管理继续拓展。“四城”联创向县域延伸，城乡环境整体提升。综合治理机场路等沿线环境，完成世纪大道等细部整理，城区环卫、清雪、市政养护等机械化作业率提高到70%。推进村容村貌“六整治、四提升”，杜尔伯特当奈村等5个村屯被国家列为“美丽乡村”建设试点。生态环境持续优化。城区植树1000万株，建成区绿化覆盖率45.3%，农村新造林15.3万亩，治理“三化”草原20.3万亩，改善龙凤湿地等水环境，整顿污染企业66家，淘汰黄标车2256台，PM监测覆盖市区。省级生态县区增加到6个、生态乡镇增加到49个。环保部城市空气质量排名，大庆紧随三亚、湛江两个沿海城市列全国第三位，居内陆城市首位。

（四）改革开放成果更多惠及百姓生活和企业发展。重点改革扎实推进。新社区管理体制全面实施，扁平化管理下沉到社区，综合服务集中在社区，新建改建社区服务综合体37个、总数达到41个，“一刻钟社区服务圈”配套功能不断提升；文化院团改制焕发新活力，《鹤鸣湖》等精品剧目市场化演出158场，大庆歌舞团走出国门赴美国拉斯维加斯驻场演出；县级公立医院改革试点稳步推进，基本药物制度实现全覆盖；3021户企业纳入“营改增”试点、减税7614万元，1.6万户小微企业暂免征收增值税和营业税；农村改革继续深化，发展农业新型经营主体1260个，土地规模经营面积达到275万亩。政府职能和作风有所转变。市级审批事项调整取消113项、下放62项。居民出入境审批由市公安局统一办理下放到各分局，市、区属地化城管体制进一步理顺；以解决价值观和作风问题为目的的政务文化建设工程继续推进，严格执行中央八项规定、省委省政府和市委有关规定，周五调研日、现场办公和集中会办等制度促进了工作方式改进和效能提升，减少政府会议20%、文件26%、公务接待35%，市本级“三公”经费支出减少22%。城市对外影响力进一步提升。综合分析资源要素、产业基础、市场潜力和关联成长性等因素，研究梳理出5大领域25条重点产业链，并在香港、深圳和广州等地成功推介，60个商协会200多位企业家分批回访，增进了对大庆了解，达成合作意向36个、签约项目15个；瑞典、以色列等5个国家25位驻华使馆代表访问大庆，推动比利时弗兰德斯汽车研发检测中心等合作项目取得新进展；《第一财经周刊》综合GDP、人均收入、一线品牌进驻密度、全球500强进入数量、大公司重点战略城市排名、机场吞吐量等10项指标，评定大庆进入全国二线城市。

　　（五）成功抵御特大洪涝灾害。2013年，松嫩两江流域遭受1998年以来最大洪水，同时乌双两河客水50年一遇，肇源、杜尔伯特和林甸的抗洪排涝形势异常严峻。面对重大自然灾害，我们坚持“指挥有力、组织有序、处置有效”，市县区合力、军地企协同、人财物联动，加固堤坝101公里，妥善处置重大险情100多处、400多次，安全转移群众9218人，人员无一伤亡，国堤无一处溃坝。同步跟进卫生防疫、水环境监测等措施有效防范次生灾害，通过补贴增购收获机械、集中组织抢收、及时修复损毁圈舍等办法，挽回农牧业损失10亿多元。及时组织受灾群众安置、倒损房屋修建、基础设施恢复等重建工作，省、市、县区累计投入重建资金3.98亿元，除1921户自愿在今年修复倒损房屋外，余下的13377户抢在去年11月中旬前全部修建完成，保证了所有受灾群众安全越冬、妥善安置。

　　各位代表，过去一年取得的成绩来之不易，这得益于市委的坚强领导，得益于人大、政协的有力监督，得益于各民主党派、工商联、无党派人士和群团组织的积极参与，得益于石油石化大企业及驻庆中省直单位的鼎力支持，得益于驻庆部队和武警官兵的无私奉献，得益于老领导、老同志的关心帮助，得益于全市广大干部群众的团结奋斗。在此，我代表市政府，向全市人民，向所有为大庆发展做出贡献的同志们、朋友们，表示崇高的敬意和衷心的感谢！

我们在看到成绩的同时，也清醒地认识到存在的矛盾和问题。主要是：接续产业基础仍不牢固，占比超过经济总量5%的支柱产业较少，产业链条较短，战略新兴产业规模较小，现代服务业比重较低，产业结构需要进一步优化；城乡二元结构依然明显，尤其在基础设施、公共服务等方面差距较大。伴随建成区面积增加、人口快速增长、机动车辆迅速增多，有些方面的“城市病”开始显现，城乡管理需要进一步加强；部分城乡居民生活水平相对较低，就业、住房、养老等方面存在的困难需要进一步解决；有些政府部门和公务人员服务意识不强，办事效率不高，存在形式主义、官僚主义、享乐主义和奢靡之风，这些问题必须高度重视、认真解决。

二、2014年工作安排

　　今年是全面深化改革的第一年，中央已做出重大决定，省委和市委都相继出台了贯彻实施意见，为我们解放思想、创新实践提供了行动指南；中央和省委加强新型城镇化建设的配套政策正密集制定出台，尤其是大庆被纳入“哈长城市群”节点城市，为我们增强城市整体影响力、提升城镇化水平注入了强劲动力；我省实施“两大平原”现代农业综合配套改革试验、黑龙江和内蒙古东北部地区沿边开发开放、大小兴安岭林区生态保护与经济转型、全国老工业基地调整改造和全国资源型城市可持续发展等五大“国字号”规划，为我们推动产业和城市进一步转型升级带来了重大机遇；大庆跻身全国二线城市，知名度和城市品牌竞争力大幅提升，多年积蓄形成的现代城市功能、产业基础后劲和生态环境优势正在加速释放，国家允许国有经济和其他所有制经济发展成为混合所有制经济、允许地方政府通过发行债券方式拓宽城市建设融资渠道等政策导向，为我们优化经济结构、破解资金瓶颈等问题开辟了新路径。所有这些都说明：我们具备“稳”的坚实基础和“进”的有利条件，必须抢抓机遇、奋发有为，在新的起点上加快大庆可持续发展。

　　今年政府工作总的要求是，深入贯彻党的十八届三中全会精神，按照市委八届四次全会部署，坚持稳中求进总基调，以改革统揽全局，以创新驱动发展，转方式、调结构、惠民生、打基础、攒后劲，力促经济和城市转型升级，加快现代化国际化城市建设步伐。主要预期目标：地区生产总值增长9%，地方财政收入增长15%，固定资产投资增长18%，社会消费品零售总额增长15%，城镇居民人均可支配收入、农民人均纯收入分别增长12%和15%。

　　（一）构建现代产业体系，推动经济持续健康发展。着眼打牢产业基础，加快构筑高新技术先导区、现代服务业核心区、城市近郊功能区、绿色生态示范区和现代农业引领区，深入实施“5·10·15”计划，力争三次产业分别增长11.6%、7.4%和16%，进一步提高“五个比重”，切实增强经济发展的质量和效益。抓好产业链生成上项目，壮大优势产业。在全力支持油田4000万吨稳产、扩大天然气产量的同时，依托资 源引入开发要素、用好存量促进对外合作、创新技术升级产业层次，新建续建投资超千万元产业项目240个以上，计划当年完成投资350亿元以上。突出拉长石化产业链，支持石化公司120万吨乙烯逐步达产、千万吨炼油二次加工项目实施，支持炼化公司与开发区合作建设15万吨丙烯项目，支持中蓝石化建设催化烟气脱硫项目，抓紧推进550万吨重油催化热裂解等项目，推动初级化工产品多次加工增值，拉长乙烯、丙烯、C等链条，向下游塑料、橡胶、聚酯等中间产品延伸，并努力实现市场终端产品落地承接。突出延伸食品产业链，重点搞好玉米、杂粮杂豆、乳制品、马铃薯、肉制品深加工，增强伊利、博润、百森、展华、雨润等企业带动力。突出做大汽车产业链，促进沃尔沃汽车规模生产，抓好鑫龙江客车和凯山、永胜等专用车项目，引进重庆江森等配件生产企业，通过整车生产带动研发体系、零部件和汽配物流关联发展。突出拓展装备制造产业链，支持油田装备企业发展，推动力神泵业、大丰科技等骨干企业增强实力，引建远景风力发电机设备生产等项目，重点发展勘探、钻井、采油、集输等石油石化装备和环保、新能源装备。突出打造新材料产业链，做好铝型材、铝箔材和半导体材料延伸加工，确保忠旺新型铝材项目达到设备安装条件、200MW恒基晶硅电池等项目投产，支持同禹傲思、佳昌科技等企业升级发展。坚持粮食、果蔬、畜牧“三位一体”，大力发展现代农业。实施两江堤防达标工程，有序推进连环湖水域整治和林甸涝区治理工程，整体提高防洪排涝抗旱能力。搞好沿江300万亩水田规划开发，建设200万亩现代农业示范区，农业机械化作业率达到90%，粮食总产135亿斤；建好大同航天果蔬、林甸润琦果蔬、红岗北极花谷等基地，果蔬产量40亿斤、花卉660万株，销售收入达到56亿元；推进畜牧养殖“三退三进”由奶牛向各类畜禽覆盖，新改扩建标准化养殖小区100个，规模化养殖比重提高到70%，肉蛋奶和水产品总产量达到260万吨；整合提升叫响优质稻米杂粮、寒地果蔬花卉、特色畜禽水产等大庆优势产品品牌，力求安全绿色可追溯、质量标准有保障、全国市场能认同。增加有效供给，刺激引导消费，大力发展现代服务业。建好唐人中心大商麦凯乐等项目，充分释放万达、松雷等大型商业综合体高端服务功能，扩大大商、庆客隆等品牌连锁店社区覆盖，强化便民服务功能；提升汽配、机电、建材等专业市场档次规模，增强区域辐射功能；扶持酷龙图等企业拓展业务，逐步构建集交易平台、实物配送、支付结算等于一体的电子商务体系，打造业态差异化、更具带动力的现代商贸格局；完善提升黑鱼湖生态景区、连环湖温泉景区二期、林甸明道温泉、阿木塔等景区功能，发挥资源组合优势，做优生态养生游、观光采摘游、展馆文化游、雪地温泉游、民俗风情游等产品，打造黄金旅游线路，推动“四全”旅游整体升级；建设高新区物流园、大同农产品物流园等项目，放大新肇内陆港等铁海联运功能，加快建设现代物流体系；以“智慧大庆”建设为牵动，推动信息资源优化整合，建设新华08（大庆）产权交易所、联想科技城、勒卡斯云计算数据中心等项目，壮大新一代信息技术产业规模。增强园区承载功能，带动关联产业和发展要素集聚。坚持高新区示范引领，经开区创新升级，各专业园区特色发展，大力完善园区道路、给排水、污水处理等基础设施，配套检验检测、投融资、供应链等专业化集成服务，园区投资强度和土地利用率提高2个百分点以上。大力引进培养领军型、创意型、技术型研发团队和高端人才，支持福瑞邦、大丰科技、佳昌等行业龙头企业与高校和科研院所合作，组建生物医药、装备制造、新材料等产业技术创新联盟，转化落地科研成果突破100项，培育发展科技型中小企业200家以上。 （二）积极稳妥推进新型城镇化，统筹建好宜居宜业家园。着眼打牢市政基础，坚持“以人为本、优化布局、生态文明、传承文化”的基本原则，科学编制大庆新型城镇化规划，把握好正确的方向、规划的实施与合理的工作节奏，释放主城区辐射带动功能，推动外围组团和新农村建设协同发展。抓好城镇布局和形态优化。市区重点推动近郊镇与主城区深度融合，稳妥有序推进“城中村”整体改造，搞好利民苑、八百垧松林小区等老居住区综合改造，持续优化城区环境；外围重点提升城关镇、做强中心镇，加快发展高台子、新站、朝阳沟等省级示范镇，加速建设四季青、连环湖、花园等新兴城镇，增强县域城镇多点辐射和经济多元支撑能力；实施“美丽乡村”建设工程，重点抓好龙凤向阳村、肇州实现新村、肇源东义顺村、林甸旭日村、杜尔伯特当奈村等国家“美丽乡村”试点，加快打造一批有历史记忆、地域特色、民族特点的美丽村庄。抓好功能设施均衡配套。围绕庆南、庆北、庆东、庆西新城开发，新建续建87个重点项目，规划推进乙烯农场、东西两站站前等区块整体开发，建好北湖国际社区和绿地国际生活乐园等项目，新建东城第二污水处理厂、餐厨废弃物处理厂等市政设施，升级改造让区龙东路、林甸外环等城乡道路，维修加固公园桥等3座桥梁，推动萨尔图机场增开航线和铁路东西两站、哈大齐铁路客运专线加快建设，抓紧建设西城长途客运枢纽和城市公交总站，实现铁路西站“零换乘”功能；完善配套乡镇和村屯基础设施，搞好巷道铺装、亮化绿化、人畜分离等综合环境改造，争取有条件的村屯尽早实现巷道有路灯、娱乐有场地、排水有通渠、垃圾有集运、房前屋后有绿带，让村屯环境好起来、晚间亮起来。抓好城乡综合治理。深入开展“四城”联创，统筹实施“三四五六”工程，全力抓好全国文明城市第三轮复检，扎实推进国家园林城市创建。持续开展城乡环境“十项”综合整治，加大公共聚集场所、城乡结合部、建筑工地等重点部位治理力度；发挥好数字城管平台作用，加密城管和公安并用的智能监控系统、新上探头3000个以上；推进环卫保洁、市政养护等装备升级，城区机械化作业率提高到75%；强化规划建设管理领域突出问题治理，严格审批监管和质量管控，落实项目法人建筑质量终身负责制；开展城区地下管网系统登记普查，并制定实施维护、改造、升级行动计划，促进地上地下协调安全发展。

　　（三）保障和改善民生，提高社会治理水平。着眼打牢社会基础，按照中央“守住底线、突出重点、完善制度、引导舆论”要求，继续深入实施“百项民生工程”，落实好具体民生实事。促进更高质量就业。推行“先照后证”放宽公司注册登记制度，落实小额担保贷款和结构性减税等政策，支持自主创业1万人以上；对城镇困难群体、农村转移劳动力和退役军人等重点人群实行就业援助；促进高校毕业生与装备、汽车、信息、食品等重点产业用工需求“订单式”对接，驻庆高校毕业生就业率达到85%以上；通过签订劳动合同，约束劳资双方促进稳定就业，城镇登记失业率控制在4.5%以内。完善基本保障。社会保险“安全网”重点向非公有制企业用工、农村居民等人群覆盖，建立社保关系便利衔接机制，统一市区新农合报销标准，“五险”新增9.7万人；推进公积金缴存向个体工商户和进城务工人员扩面；深入落实城市“十位一体”、农村“六位一体”低保综合救助，按照低保标准1.5倍认定低收入家庭并落实临时救助政策；建设保障性住房5600套，在推进廉租房和公租房并轨、保证供应量的同时，探索“共有产权保障房”模式，帮助收入稳定、暂时买不起商品房又渴望拥有自己住房的人群实现“有房梦”。完善公共服务。着眼破解教育资源均衡配置难题，利用现代技术手段，实施“优质教育资源共享工程”，突出完善提升远程教育系统，今年硬件终端延伸到乡镇学校，实现名师网络在线实时授课、互动教研，让学生能够在线享受到实景式课堂教学服务。建立市级教学资源数据库，录制名师教学课件，配建中小学校多媒体教学设备，逐步使所有学校都能通过视频播放体验市内外优秀教师的教学实况；新增义务教育标准化学校10所、标准化率提高3个百分点，新建公办幼儿园15所，扶持民办园，整改不规范园，建设大庆师范学院附属幼儿园并发挥其学科优势，使之成为全市学前教育实习实训基地和示范培训基地。特色化发展职业教育，突出培养装备、汽车、食品、旅游和信息等重点产业的专业实用人才；推进乡镇卫生院和村卫生室一体化管理，继续配套城乡基层医疗卫生服务机构设施设备。油田总医院、市人民医院等综合医院牵头实施乡医全覆盖培训计划。支持市中医院实施国家中医药预防保健及临床服务项目。引进解放军301医院等国内顶级医院培训项目，拓展“开放办医”成效；坚持公办养老机构保基本，通过“公建民营”和“民建公助”的方式引导社会兴办养老机构，新增床位1000张。支持大企业所属部分二级医院和社会医疗机构转型增设老年人护理、康复、临终关怀等服务项目，积极稳妥推广“医养结合”模式。完善“呼叫热线”系统，提供更周全的社区居家养老服务功能；新开通乙烯至新村、西客站至人民医院2条公交快线，将现有25家出租车公司整合为5家，新增运力1000台，实现规模经营、标准服务、有序竞争。强化社会治理。完善市民网络问政平台，抓好群众工作中心启动运营，提升市长热线受理功能，开通党政领导视频接访系统，严格落实市县乡主要领导带头的领导包保重点信访案件制度，确保信访积案化解率达到80%以上；建立健全农产品质量、食品安全追溯和质量标识制度，建设市级食品药品检验检测中心、四县和大同检验检测所，扩大食品药品基层监管覆盖；常态化、零告知排查生产安全隐患，执行隐患整改台账销号制度，重点抓好油气管线占压、危险化学品生产储运、道路交通等领域专项整治和“打非治违”专项行动，集中完善学校、幼儿园、养老机构等公共场所安全设施，确保安全生产形势平稳；深入开展“打四黑除四害”专项行动，加大网络诈骗等新型犯罪打击力度，维护社会安定。

　　（四）推动文化繁荣发展，增强城市软实力。着眼打牢人文基础，深度挖掘特色文化资源，大力推进文化惠民，做优文化产业。筑牢城市之魂。培育和践行社会主义核心价值观，传承弘扬大庆精神铁人精神和“三老四严”优良传统，筑牢大庆人文根脉；巩固大庆精神铁人精神特色国民教育的常态化和规范化建设，统筹各学段科学整体推进；深入开展道德领域突出问题专项整治，倡导“十大观念”、整改“十大陋习”，让文明根植心底、走进生活。加强公共文化服务。搞好奥体中心“一场三馆”等大型文体场馆运营，采取定期免费、长期会员合理收费等多种形式向市民开放，通过市场化运作积极承办国内外赛事和演出，办好WCBA全明星赛；拓展“文化大篷车”等惠民工程，开展好专业文艺团队基层巡演和广场舞等群众性文化活动；推动广播电视由“村村通”向“户户通”拓展，建设100个以上农村固定电影放映点；确保党史馆、非物质文化遗产展示馆、东北第四纪古生物化石国家研究保护中心等项目建成投用。壮大文化产业。建好文化创意产业园，完善黑龙江国际艺术村、百湖艺术群落等项目功能，加快湿地文化公园建设，壮大新闻传媒集团、文体旅集团、油田文化集团等骨干企业实力。创意更多“大庆元素”文化产品，推动 文化与旅游、观光农业、时尚产业等关联融合发展。引导形成社会健康心态。更加注重人文关怀，突出网上舆情疏控，及时准确回应社会关切，传导积极心态，调适消极心态，矫正非理性心态，持续为建设现代化国际化城市增添正能量。

　　（五）推动全面深化改革，扩大对外开放。着眼打牢体制基础，最大限度增强发展活力，在市委领导下“正确、准确、有序、协调”推进各项改革。抓好改革任务落实。按照中央和省委全面深化改革的要求，认真落实《中共大庆市委关于贯彻落实党的十八届三中全会精神全面深化改革的实施意见》和今年8个方面重点改革任务。注重强化责任，健全工作机制，搞好调查研究，精细制定方案，确保实现准确衔接；注重把握节奏，对已经明确要求的改革不等不拖，正在研究出台的做好准备，允许探索尝试的大胆实践，确保时机和力度精准；注重各方联动，有效引导舆论，全程督查督办，确保改革协调有序推进。推进政府职能转变。履行好“公共服务、市场监管、社会管理、环境保护”地方政府职责，进一步下放事权，国家和省里要求取消下放的行政审批项目坚决取消下放，没有法律法规依据和变相保留的行政审批项目坚决取消，依据我市情况应该和可以取消的行政审批项目坚决取消；进一步放开市场，梳理政府“权力清单”，对保留的行政审批事项编制目录，优化流程设计，并全部对社会公开。梳理确定市场“负面清单”，实行“非禁即入”；进一步提高治理能力，坚持多元参与共治、基层组织自治、多种手段综治，重点培育发展社会组织，积极发展行业协会、中介机构、志愿团体，引导社会各界和公众有序参与公共事务管理。顺应改革大势探索创新。深化户籍制度改革，有序放开落户限制；加快发展混合所有制经济，重点探索地企合作新途径，争取在小油田开发、石化延伸加工以及装备、物流等领域率先突破；深化市属国资国企改革，完善法人治理结构，支持已具备资本投资功能的市属企业改组为国有资本投资公司；推动农业综合配套改革，重点培育专业大户、家庭农场、农民合作社等新型农业经营主体，推动农村信用社改制为农村商业银行，促进有条件的小额贷款公司转制为村镇银行；引导社会资本通过BOT等特许经营模式参与城乡基础设施建设和运营；进一步完善工程招投标制度，健全工程建设各环节责任约束机制，严格规划、立项、施工、验收、决算、审计等程序，做到全程依法依规完整衔接。进一步扩大对外交流合作。打消大庆不沿边不沿海的顾虑，按照交易活动、货物通关、产品制造“三地分离”原则，在对外合作特别是对俄开放开发中主动站位、扬长避短，坚持“出口抓加工、进口抓落地”，重点在园区合作共建、能源合作开发和食品、汽车、生物医药等产业项目合作上突破。继续深化与香港、深圳、广州等地商协会合作，抓好产业链招商，集聚引进外埠发展要素，确保招商引资额持续增加。拓展对外经贸合作，新增外贸企业30家，外贸进出口总值增长20%。

　　（六）抓好生态文明建设，推动绿色低碳可持续发展。着眼组合放大生态新优势，让生态魅力转化为竞争实力。引导全社会践行生态价值观。突出政府主导意识，谋发展必须考虑生态承载能力，上项目严格执行生态环保安全准入标准，实行政府相关责任人自然资源资产离任审计，推动形成经济增长和生态优化协调共进的理政价值取向；突出企业主体责任意识，遵守环保相关法律规定，自觉做到清洁生产、达标排放，让生产区变成生态区；突出民众主人翁意识，倡导低碳绿色生活方式，自觉摒弃影响生态环境的不文明行为，使生态文明建设为众人、集众智、靠众行。继续实施好生态环境保护工程。以创建国家园林城市为牵动，深入推进植树、复草、治水、净气、降噪“五大工程”，建好森林植物园等生态景观项目，拓展油田矿区“百里绿色廊带”，继续抓好城区绿化、农村造林和“三化”草原改良，进一步改善龙凤湿地等水体环境，地企共同保护和治理龙虎泡水源地；强化工业污染源监控，严格企业排污监管，开展汽车尾气、餐饮油烟等专项整治，努力保障PM指标持续控制在优良水平。建立完善严格的生态文明制度体系。健全自然资源资产产权制度和用途管制制度，对河湖、森林、滩涂、草原、荒地等自然生态空间统一确权登记，完善滨水区域开发与保护办法，严厉打击非法倒包、出租、破坏生态资源等行为；建立生态投入长效机制，探索吸引社会资本投入生态环境保护的市场化模式；落实节能减排责任制，万元地区生产总值综合能耗下降3.3%，化学需氧量削减2150吨。放大生态综合效益。在保护好生态的前提下，依托优质空气、湖泊、湿地、草原、温泉、冰雪等生态资源组合优势，契合自然颐养、健康服务等新兴时尚需求，创意催生“候鸟式”休闲度假、养老养生等产业，用生态资本换来环保资产。

　　各位代表，完成全年发展任务目标，必须坚持市委统一领导，主动接受人大法律监督和工作监督，自觉接受政协民主监督，广泛听取各界意见和建议，按照市委开展党的群众路线教育实践活动总体部署，结合全面深化改革要求，突出问题导向，切实抓好政府自身建设。着重在改进作风上下功夫。严格执行中央八项规定、省委省政府九项规定、市委十个方面37条要求，以及近期中央相继出台的厉行节约反对浪费、公务接待管理、“三公”经费使用、差旅费管理等相关规定，守住纪律和法律红线、底线。聚焦“四风”，深入查摆整改形式主义、官僚主义、享乐主义和奢靡之风等方面存在的突出问题。坚持“负责任地干事，干负责任的事”理念，深入抓好政务文化建设工程，把作风改进成果上升到制度层面，转化为文化力量，形成行为自觉。着重在保持良好状态上下功夫。克服精神懈怠、思想畏难等萎靡情绪，增强责任担当意识，保持工作热情和创业激情；克服“少干事没啥事、多干事怕出事”等消极心态，用心想事、主动干事、努力成事；克服韧劲不足、不敢碰硬等虚浮行为，发扬“钉钉子”精神，埋头实干、持之以恒，确保每件事抓出头见实效。着重在依法行政上下功夫。强化法治意识和法律观念，政府一切工作坚持有法必依、执法必严、违法必究，尤其对食品药品、安全生产、环境保护、劳动保障等重点监管领域严明责任、严格执法、严肃整治，营造放心安全的发展环境。着重在提升效能上下功夫。针对政策、规定、制度和程序分散在各部门带来的不便了解等问题，编制行政服务指南“一本通”，让企业和群众办事更便捷；针对深入基层一线力度不够等问题，落实好周五调研日制度，坚持现场办公和集中会办常态化，确保困难问题及时发现和解决；针对工作中因“中梗阻”落实不到位等问题，深入开展“双十行动”，严厉查处庸懒散奢、吃拿卡要、推拖躲绕等行为，务求干部清正、政府清廉、政治清明。

　　各位代表，落实改革任务需要攻坚克难，实现发展目标需要奋发有为，满足群众期待需要倾心倾力。让我们在市委的坚强领导下，团结依靠全市人民，弘扬大庆精神、铁人精神，解放思想、与时俱进、居安思危、团结拼搏，为加快建设现代化国际化城市而努力奋斗！
